# Comparison of treatment outcomes for native tissue repair and sacrocolpopexy as apical suspension procedures at the time of hysterectomy for uterine prolapse

**DOI:** 10.1038/s41598-021-82732-0

**Published:** 2021-02-04

**Authors:** Sumin Oh, E. Kyung Shin, Sowoon Hyun, Myung Jae Jeon

**Affiliations:** 1 Department of Obstetrics and Gynecology, Seoul National University Hospital, Seoul, 03080 Republic of Korea; 2 Department of Obstetrics and Gynecology, Seoul National University College of Medicine, Seoul, 03080 Republic of Korea

**Keywords:** Health care, Medical research

## Abstract

Concomitant apical suspension should be performed at the time of hysterectomy for uterine prolapse to reduce the risk of recurrent prolapse. Native tissue repair (NTR) and sacrocolpopexy (SCP) are commonly used apical suspension procedures; however, it remains unclear which one is preferred. This study aimed to compare the treatment outcomes of NTR and SCP in terms of surgical failure, complication and reoperation rates. Surgical failure was defined as the presence of vaginal bulge symptoms, any prolapse beyond the hymen, or retreatment for prolapse. This retrospective cohort study included 523 patients who had undergone NTR (n = 272) or SCP (n = 251) along with hysterectomy for uterine prolapse and who had at least 4-month follow-up visits. During the median 3-year follow-up period, the surgical failure rate was higher in the NTR group (21.3% vs 6.4%, *P* < 0.01), with a low rate of retreatment in both groups. Overall complication rates were similar, but complications requiring surgical correction under anesthesia were more common in the SCP group (7.2% vs 0.4%, *P* < 0.01). As a result, the total reoperation rate was significantly higher in the SCP group (8.0% vs 2.6%, *P* = 0.02). Taken together, NTR may be a preferred option for apical suspension when hysterectomy is performed for uterine prolapse.

## Introduction

Uterine prolapse is one of the major indications for hysterectomy; 11–13% of hysterectomies are performed to treat uterine prolapse^1,2^. However, hysterectomy alone is not adequate, and concomitant apical suspension should be performed to reduce the risk of recurrent prolapse^3–5^. Native tissue repair (NTR) and sacrocolpopexy (SCP) are commonly used; however, it remains unclear which one is the preferred option as a concomitant apical suspension procedure at the time of hysterectomy for uterine prolapse.

A recent Cochrane systematic review of six randomized controlled trials demonstrated that NTR is associated with a higher risk of prolapse recurrence and repeat surgery for prolapse than SCP, with a shorter operating time being the only advantage^6^. Another systematic review that included large case-series and comparative studies (both randomized and nonrandomized) also showed results that favored SCP over vaginal NTR in terms of anatomic success. However, there was no difference in the reoperation rates, and adverse events such as thromboembolism, ileus or small bowel obstruction, and mesh or suture complications were more frequent after SCP^7^. SCP is considered the gold-standard procedure for treating apical vaginal prolapse. However, given the inconsistent results regarding the issue of safety, we cannot draw a conclusion that SCP is better than NTR. Moreover, these systematic reviews primarily included cases of vaginal vault prolapse, and the results could not be directly applied to cases of uterine prolapse. Indeed, several studies have reported that mesh erosion rates after SCP might be increasing when it is performed along with hysterectomy^8–12^.

The primary aim of this study was to compare NTR and SCP as apical suspension procedures at the time of hysterectomy for uterine prolapse in terms of the reoperation rate for prolapse recurrence and complications. We hypothesized that the total reoperation rate would be lower in the NTR group than the SCP group. Secondary aims included perioperative outcomes, surgical failure, and complications.

## Results

The median follow-up time in both groups was 36 months (range 4–120), with no difference between the two groups (*P* = 0.49). Table 1 presents the baseline characteristics of the study population. There were differences between the two groups with respect to age and POPQ stage (*P* < 0.01); patients in the SCP group were younger and had more advanced prolapse. Patients in the SCP group had a lower rate of concomitant procedures performed to correct other compartmental prolapse; however, patients in this group were associated with a longer operating time and hospital stay, and more loss of blood than patients in the NTR group (*P* < 0.05) (Table 2).

**Table 1:** Baseline characteristics of the study population. Data are presented as the median (interquartile range) or number (%). NTR, native tissue repair; POPQ, pelvic organ prolapse quantification; SCP, sacrocolpopexy.

Variable	NTR (N = 272)	SCP (N = 251)	*P*-value
Age (years)	70 (59–81)	64 (52–76)	< 0.01
Vaginal parity	3 (1–5)	3 (2–4)	0.43
Body mass index (kg/m^2^)	24.8 (20.9–28.7)	24.3 (20.7–27.9)	0.24
Current smoker	1 (0.4)	0	1.00
Diabetes mellitus	56 (20.6)	40 (15.9)	0.17
Prior surgery for prolapse (anterior or posterior repair)	5 (1.8)	10 (4.0)	0.14
Prior surgery for incontinence	13 (4.8)	16 (6.4)	0.43
**POPQ stage**			
2 3 4	69 (25.4) 180 (66.2) 23 (8.5)	34 (13.5) 175 (69.7) 42 (16.7)	< 0.01

**Table 2:** Concomitant procedures and perioperative outcomes. Data are presented as the median (interquartile range) or number (%). NA, not applicable; NTR, native tissue repair; SCP, sacrocolpopexy.

Variable	NTR (N = 272)	SCP (N = 251)	*P*-value
**Concomitant procedures**			
Total hysterectomy Anterior repair Posterior repair Transobturator tape	272 (100) 181 (66.5) 218 (80.1) 94 (34.6)	251 (100) 0 121 (48.2) 114 (45.4)	NA < 0.01 < 0.01 0.01
**Perioperative outcomes**			
Operating time, min	145 (96–194)	200 (145–255)	< 0.01
Estimated blood loss (mL)	150 (0–300)	200 (20–380)	0.04
Length of hospital stay (day)	5 (3–7)	7 (5–9)	< 0.01

Table 3 displays the prolapse treatment outcomes. The surgical failure rate was significantly higher in the NTR group than in the SCP group (21.3% vs 6.4%, *P* < 0.01). The NTR group had more cases of symptomatic and anatomic recurrence (*P* < 0.01). However, retreatment rates were low in both groups, with no significant difference between the groups (*P* = 0.09).

**Table 3:** Prolapse treatment outcomes. Data are presented as number (%). CI, confidence interval; NTR, native tissue repair; OR, odds ratio; SCP, sacrocolpopexy. ^a^Adjusted for age and preoperative POPQ stage. ^b^Symptomatic or anatomic recurrence or retreatment for prolapse. ^c^Presence of vaginal bulge symptoms defined as an affirmative response to question 3 on the PFDI-20. ^d^Presence of any POPQ point beyond the hymen.

Variable	NTR (N = 272)	SCP (N = 251)	OR (95% CI)^a^	*P*-value^a^
Surgical failure^b^	58 (21.3)	16 (6.4)	4.65 (2.49–8.67)	< 0.01
Symptomatic recurrence^c^	36 (13.2)	11 (4.4)	4.05 (1.93–8.51)	< 0.01
Anatomic recurrence^d^ Ba > 0 C > 0 Bp > 0	53 (19.5) 53 (19.5) 0 0	14 (5.6) 4 (1.6) 0 10 (4.0)	4.50 (2.34–8.64)	< 0.01
Retreatment for prolapse Pessary insertion Reoperation	12 (4.4) 6 (2.2) 6 (2.2)	2 (0.8) 0 2 (0.8)	3.97 (0.82–19.31)	0.09

Overall complication rates were similar between the two groups except for pulmonary and gastrointestinal complications; they were more common in the SCP group, mainly owing to the presence of atelectasis and ileus (*P* < 0.01). However, complications that required surgical intervention under anesthesia (Clavien–Dindo grade IIIb) were more common in the SCP group (0.4% in the NTR group vs 7.2% in the SCP group, *P* < 0.01). Most reoperations were performed to correct mesh erosion (Table 4).

**Table 4:** Complications and Clavien–Dindo grades. Data are presented as number (%). ARDS, acute respiratory distress syndrome; CI, confidence interval; NA, not applicable; NTR, native tissue repair; OR: odds ratio; SCP: sacrocolpopexy. ^a^Adjusted for age and preoperative POPQ stage. ^b^Includes sigmoid colon perforation detected at postoperative day 8. ^c^Includes vesicovaginal fistula (n = 1), incisional hernia (n = 2), and vaginal mesh erosion (n = 15).

Variable	NTR (N = 272)	SCP (N = 251)	OR (95% CI)^a^	*P* value^a^
**Cardiovascular (total)** Hemorrhage/hematoma Thromboembolism Myocardial infarction Congestive heart failure Arrhythmia	2 (0.7) 0 0 0 0 2 (0.7)	8 (3.2) 6 (2.4) 2 (0.8) 0 0 0	0.26 (0.05–1.35)	0.11
**Pulmonary (total)** Atelectasis Pulmonary edema Pneumonia ARDS	3 (1.1) 3 (1.1) 0 0 0	14 (5.6) 9 (3.6) 3 (1.2) 2 (0.8) 0	0.15 (0.04–0.57)	< 0.01
**Gastrointestinal (total)** Bowel injury Fistula Ileus Small bowel obstruction	1 (0.4) 1 (0.4) 0 0 0	8 (3.2) 0 0 8 (3.2) 0	0.04 (0.00–0.39)	< 0.01
**Urinary (total)** Bladder injury Fistula Ureteral obstruction Urinary tract infection	38 (14.0) 0 0 2 (0.7) 36 (13.2)	31 (12.4) 0 1 (0.4) 0 31 (12.4)	1.07 (0.61–1.85)	0.82
**Wound (total)** Incisional hernia Dehiscence Infection Granulation Suture erosion Mesh erosion	46 (16.9) 0 0 21 (7.7) 26 (9.6) 3 (1.1) –	47 (18.7) 2 (0.8) 8 (3.2) 15 (6.0) 13 (5.2) 0 20 (8.0)	0.94 (0.58–1.52)	0.94
**Neurological (total)** Buttock or low back pain Lower extremity neuropathy	15 (5.5) 12 (4.4) 4 (1.5)	4 (1.6) 3 (1.2) 2 (0.8)	2.23 (0.67–7.37)	0.19
**Clavien–Dindo grade**				
Any I II IIIa IIIb IV–V	96 (35.3) 18 (6.6) 72 (26.5) 13 (4.8) 1 (0.4)^b^ 0	93 (37.1) 18 (7.2) 65 (25.9) 9 (3.6) 18 (7.2)^c^ 0	0.88 (0.60–1.29) 0.59 (0.28–1.27) 1.04 (0.68–1.58) 1.17 (0.46–2.98) 0.05 (0.01–0.40) NA	0.51 0.18 0.86 0.75 < 0.01 NA

**Figure 1:**
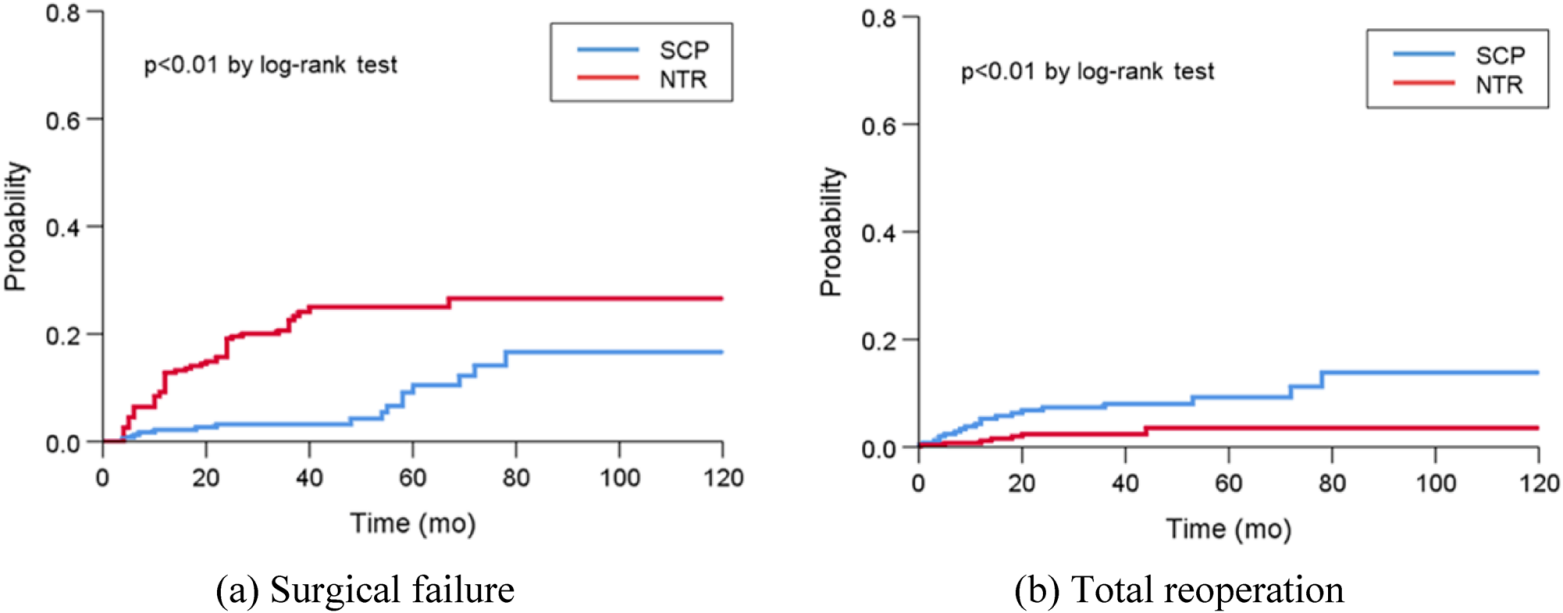
Kaplan–Meier survival curves for (**a**) surgical failure and (**b**) total reoperation for prolapse recurrence and complications. NTR, native tissue repair; SCP, sacrocolpopexy.

As a result, the total reoperation rate for prolapse recurrence and complications was significantly higher in the SCP group than in the NTR group (8.0% vs 2.6%, *P* = 0.02). Figure 1 displays the Kaplan–Meier survival curves for the occurrence of surgical failure and total reoperation. The time to surgical failure was significantly shorter in the NTR group, whereas the time to total reoperation was significantly shorter in the SCP group (*P* < 0.01 by log-rank test).

## Discussion

In the present study, we evaluated the treatment outcomes of NTR and SCP as apical suspension procedures at the time of hysterectomy for uterine prolapse. Although SCP had a lower risk of symptomatic and anatomic prolapse recurrence than NTR, the retreatment rate for prolapse recurrence was not significantly different. In addition, SCP was associated with a longer operating time and hospital stay and more blood loss, and the reoperation rate for complications and total reoperation rate (including prolapse recurrence and complications) were significantly higher in the SCP group. The majority of reoperations were due to mesh erosion.

There is a wide variation in the reported surgical failure rates depending on the definition used. We used a clinically relevant criterion recommended by the National Institute of Child Health and Human Development (NICHD) Pelvic Floor Disorders Network to determine surgical failure^13^. Our results were consistent with the findings of a recent study by Rogers et al^14^. They retrospectively compared surgical failure rates after NTR and SCP using the data from three multicenter randomized trials conducted by the NICHD Pelvic Floor Disorders Network (the Colpopexy and Urinary Reduction Efforts [CARE], Outcomes following Vaginal Prolapse Repair and Midurethral Sling [OPUS], and the Operations and Pelvic Muscle Training in the Management of Apical Support Loss [OPTIMAL] trials) and the same definition as ours. Two years after surgery, the surgical failure rate was significantly higher in the NTR group than in the SCP group (27% vs 11%). The retreatment rates for prolapse were low in both groups (5% in the NTR group vs 2% in the SCP group). However, contrary to our results, there was no significant difference in the rates of serious adverse events. This discrepancy might be explained as follows. The major complication that required reoperation in our SCP group was mesh erosion. While all women underwent total hysterectomy in our study, only half of the women underwent total hysterectomy in the Rogers et al.’s study. Concomitant total hysterectomy can increase the risk of mesh erosion after SCP up to seven-fold^10^, and this difference in the rate of concomitant total hysterectomy might affect the outcome.

Notably, surgical failure after NTR and SCP had a different pattern in our study. Despite a progressive increase over time, most cases of surgical failure after NTR occurred within the first two years. All cases of anatomic recurrence involved the anterior vagina. On the other hand, the surgical failure rates after SCP abruptly increased four years after surgery, and most cases of anatomic recurrence involved the posterior vagina. This is in agreement with the long-term follow-up findings of the CARE and OPTIMAL trials^15,16^. The reason for the increase in late-onset prolapse recurrence in the posterior vaginal compartment after SCP is not clear, but it might be related to the surgical technique used for mesh fixation. We secured the anterior and posterior leaves of the mesh to the proximal 3 cm of the vaginal cuff, which could provide durable support for the vaginal apex and upper anterior and posterior vagina, possibly making the uncovered vaginal area vulnerable to loading forces. In addition, the proximal arm of the Y-shaped mesh was secured to the anterior longitudinal ligament of the sacrum at or just below the level of the sacral promontory. This technique helps reduce the risk of hemorrhage from the sacral venous plexus but may expose the posterior vaginal compartment to loading forces by deviating the vaginal axis forward^17^.

Overall complication rates did not differ between the groups, and serious adverse events were uncommon in both groups. The majority of patients with complications responded to conservative therapy; however, 36% of the patients with wound complications in the SCP group finally required surgical correction under anesthesia. There were cases of two incisional hernia in our study. One was detected in the 10-mm trocar site at postoperative day 5, possibly related to inadequate fascial closure, and was immediately corrected with manual reduction of the herniated sac and suture repair. The other was detected in the lower midline incision site at postoperative month 8 and was repaired using mesh at postoperative month 20. The incisional hernia rate after laparotomic SCP is reported to be 5% (range 0.4–15) in the literature, with increasing rates over time^17^. Several factors, including previous laparotomy, obesity, smoking, chronic respiratory disease, and poor tissue quality, increase the risk of incisional hernia^18^, and a midline incision may also increase the risk compared with Pfannenstiel’s incision^19^. We used a Pfannenstiel approach in almost all cases of laparotomic SCP unless there was a previous midline scar. This may explain why the incidence of incisional hernia was low (0.8%) in our patients despite long-term surveillance. Nonetheless, the mesh erosion rate after SCP was high (8.0%) in our study, considering the overall rate of 3.4% reported in a systematic review^17^. Although smoking and the use of non-type 1 polypropylene mesh are known to increase the risk of mesh erosion^8^, this was not the case in our study; there were no smokers, and we used type 1 polypropylene mesh (Gynemesh PS) in all cases. Two-thirds (13/20) of mesh erosion cases were detected at the vaginal cuff within four months after surgery in our study. Gynemesh PS is known to induce strong foreign body inflammatory responses to the mesh insertion site, and excessive and prolonged release of matrix metalloproteinases can destroy collagen and elastin, which may result in poor healing at the cuff after hysterectomy^20^. This may explain why the mesh erosion rate after SCP was high in spite of the use of type 1 polypropylene mesh in our patients. A recent long-term follow-up study of laparoscopic SCP using polypropylene mesh with similar weight to Gynemesh also reported that the mesh erosion rate was higher after hysterectomy with SCP than after sacrohysteropexy (7.3% vs 3.7%)^21^.

The strengths of our study include a large study population. Our cohort study also benefited from a long-term follow-up period, the use of a clinically relevant criterion to define surgical outcome, and detailed perioperative and postoperative information.

Nonetheless, there are some limitations, which are mainly attributable to the inherent weaknesses of a retrospective study. There were some differences in the baseline characteristics between the two groups, reflecting a selection bias (i.e., patients with risk factors for prolapse recurrence, including young age and advanced prolapse, were more likely to have received SCP that could provide a durable pelvic support). To minimize a possible confounding effect, we used logistic regression models with adjustment for imbalanced baseline variables (age and preoperative POPQ stage) when outcome analyses were performed. All surgeries were performed by a single expert surgeon, which limits the generalization of the findings of this study. In addition, all postoperative POPQ assessments were performed by the operating surgeon, which may have resulted in an underestimation of surgical failure^22^. Nonetheless, our surgical outcomes were comparable to the findings of another study that used data from three multicenter, randomized trials conducted by the NICHD Pelvic Floor Disorders Network. The use of Gynemesh PS might have also affected our results. Although lighter type 1 polypropylene meshes have been developed, they were not available in Korea during the study period. Because lighter mesh induces weaker foreign body inflammatory responses than Gynemesh PS^20^, the use of lighter mesh may reduce mesh erosion rates after SCP^23^. However, it may negatively influence the durability of SCP, with an earlier recurrence of prolapse^24^.

The surgical decision-making process for pelvic organ prolapse is complex. It is important to provide adequate information on the risks and benefits of the available options for correcting prolapse and to guide patients’ decision-making. Although recurrence rates favor SCP over NTR, retreatment rates for prolapse are low in both groups with no significant difference. Considering the higher rate of reoperation (including prolapse recurrence and complications) after SCP, NTR may be a preferred option for apical suspension when hysterectomy is performed for uterine prolapse. A well-designed, prospective, randomized trial is needed to support our findings.

## Methods

### Patient data collection

We reviewed the medical records of 545 patients who had undergone an apical suspension procedure along with hysterectomy for pelvic organ prolapse quantification (POPQ) stage 2–4 prolapse at Seoul National University Hospital between November 2008 and April 2018. Of them, 22 patients who had a follow-up period of less than four months were excluded from the analysis. The study was approved by the institutional review board (Seoul National University College of Medicine/Seoul National University Hospital 2005-045-1122) and informed consent was waived by the institutional review board because of the nature of the retrospective study. All methods used in this study were performed in accordance with the relevant guidelines and regulations.

At baseline, all patients completed the Pelvic Floor Distress Inventory Short Form (PFDI-20) questionnaire^25^, provided a complete medical history, and underwent POPQ examination in a 45° upright sitting position with an empty bladder^26^. Among the 523 patients included in this study, 272 underwent NTR, and 251 underwent SCP for apical suspension. NTR consisted of 129 cases of iliococcygeus suspension (ICG) and 143 cases of uterosacral ligament suspension (USLS). ICG was performed transvaginally, and USLS was performed either transvaginally or transabdominally. SCP included 223 cases of laparotomic and 28 laparoscopic approaches. All operations were performed by one skilled urogynecologist (M.J. Jeon), as described in previous reports^27–29^. For SCP, we used a 10-cm × 4-cm polypropylene mesh (Gynemesh PS; Ethicon, Somerville, NJ) fashioned in a Y shape from two pieces of mesh. Patients with urodynamic stress incontinence underwent additional transobturator tape procedures at the time of prolapse surgery, as described in a previous report^30^.

Scheduled in-person postoperative follow-up visits occurred at 1, 4–6, and 10–12 months and then annually thereafter. At each visit, patients underwent a clinical examination including the POPQ and, starting from the 4–6 month visit, were asked to complete the PFDI-20. In addition, new or continuing pelvic floor disorders and adverse events that had occurred since the previous evaluation were assessed. Patients were considered to have surgical failure if they had anatomic recurrence (defined as the presence of any POPQ point beyond the hymen) or symptomatic recurrence (presence of vaginal bulge symptoms defined as an affirmative response to question 3 on the PFDI-20) or if they underwent retreatment for prolapse with either surgery or pessary insertion. Complications were classified using the Clavien–Dindo grading system^31^. Bladder and bowel dysfunction unrelated to visceral injury, complications unrelated to apical prolapse surgery such as anesthesia complications and complications that unequivocally resulted from concomitant procedures were excluded from the analyses.

### Statistical analysis

Data were analyzed using SPSS software (version 25; SPSS Inc., Chicago, IL). The normality of data was assessed using the Shapiro–Wilk test, which indicated that data did not follow a normal distribution. Therefore, comparisons between the groups for continuous variables were performed using the Mann–Whitney U test. To compare the categorical variables between the two groups, Fisher’s exact test or the chi-squared test was performed. Analyses of pelvic organ prolapse outcomes and complication data were performed using logistic regression models with adjustment for age and preoperative POPQ stage. In addition, Kaplan–Meier survival analysis was used to compare time-to-event outcomes. A *P*-value of < 0.05 was considered statistically significant.
